# Prevalence of co-morbidities among periodontitis and non-periodontitis Indian patients

**DOI:** 10.6026/973206300200888

**Published:** 2024-08-31

**Authors:** Dinesh Saini, Shivani Dhawan, Rajan Dhawan, Paras Gupta, Javvadi Kiranmayi, Rishika Singh

**Affiliations:** 1 Department of Periodontology, Maharishi Markandeshwar College of Dental Sciences and Research, Mullana, Ambala, India; 2 Department of Conservative Dentistry and Endodontics, Maharishi Markandeshwar College of Dental Sciences and Research, Mullana, Ambala, India; 3 Department of Oral Medicine and Radiology, Maharishi Markandeshwar College of Dental Sciences and Research, Mullana, Ambala, India

**Keywords:** Comorbidities, periodontitis, obesity, systemic diseases

## Abstract

Periodontitis being an immuno-inflammatory disease can act as an aggravating factor for various systemic diseases. Therefore, it is of interest to document the prevalence of comorbidities among periodontitis and non-periodontitis Indian patients. 680 patients were enrolled, categorized into non-periodontitis group (Group A) and periodontitis group (Group B). Each group was again sub-grouped into comorbidities and non-co-morbidities. Periodontitis patients were found to have significantly more co-morbidities than non-periodontitis patients. Osteoporosis and obesity showed statistically higher levels in periodontitis patients. Hypertension, diabetes mellitus, cardiovascular diseases, and COVID-19 infection showed higher prevalence in periodontitis group, though the difference was statistically non-significant.

## Background:

The connection between poor oral hygiene and entire systemic health has been an area of growing interest and research. In the age of evidence-based dentistry, researchers have started to offer a growing amount of data indicating that untreated periodontitis can have a systemic impact on a person. [1] Chronic inflammation linked with periodontitis can trigger immune responses that can contribute to the pathophysiology of various systemic diseases like diabetes mellitus, cardiovascular diseases, pulmonary diseases, cancer, and preterm low birth weight infants [2-3]. Numerous bacterial products and inflammatory mediators are stored in the periodontal pockets and the tissues that surround them. [4] These include lipopolysaccharides (LPS) and cytokines like interleukin 1, tumor necrosis factor-alpha, and prostaglandin E2. [5] The pathogens in periodontitis can give rise to systemic diseases by transient bacteremia, circulation of their toxins, and by inducing immune-inflammatory response via sulcular epithelium which is often ulcerated and discontinuous. [6] Understanding how oral inflammation affects various other organ systems and how it can raise the risk of developing inflammatory non-oral diseases, requires an insight of the relationship between systemic and gingival inflammation. [7] Therefore, it is of interest to document the prevalence of comorbidities among periodontitis and non-periodontitis Indian patients.

## Materials and Methods:

This cross-sectional study was conducted in the Department of Periodontology, Maharishi Markandeshwar College of Dental Science and Research, Mullana. The study was conducted as per the Declaration of Helsinki (1964) revised in 2013, with the approval of the Institutional Ethical Committee of Maharishi Markandeshwar (Deemed to be University), Mullana, Ambala (IEC-2279)

## Patient sample:

680 patients (381 males and 299 females) between the age of 20 to 55 years, were randomly selected from the outpatient department. Pregnant females, lactating mothers, and patients who have received periodontal treatment during the last 6 months were excluded. The patients selected were explained about the objectives of the study and informed consent was obtained before commencement of the study.

## Study design:

Patients selected were equally divided into two groups: Group A (Non-periodontitis Group) comprising 340 patients, who did not show any clinical signs of periodontitis, and Group B (Periodontitis Group) comprising 340 patients, who displayed interdental clinical attachment loss (CAL) ≥ 3mm. Each Group was sub-grouped according to associated co-morbidities and non-comorbidities.

## Clinical parameters:

All the selected patients were subjected to clinical parameters: Plaque index (PI)[8], Probing pocket depth (PPD), Papillary bleeding index (PBI)[9], Clinical attachment loss (CAL), and Body mass index (BMI).

## Questionnaire:

A questionnaire containing questions about the link of periodontal health/periodontitis with co-morbidities and COVID-19, accompanied by demographic details was given to the patients; and then evaluated for their responses. Educational and Occupational scores were evaluated according to the Modified Kuppuswamy scale. [10] All the data collected was subjected to statistical analysis.

## Statistical analysis:

"Independent Sample t-test" and "Paired Chi-square-test" were applied for statistical analysis. Significance was defined by a p-value less than 0.05 using a two-tailed test. Data analysis was performed using IBM-SPSS version 21.0 (IBM-SPSS Science Inc., Chicago, IL).

## Results:

Among patients in Group A, 22.5% of cases had comorbidities, whereas among those in Group B, 29.2% had comorbidities, as seen in Table 1. The two groups had no statistically significant difference in overall comorbidities (p=0.095). 88 comorbidities were found in non-periodontitis patients, whereas 123 were in periodontitis patients. There was a statistically significant difference in overall comorbidities between non-periodontitis and periodontitis groups (p=0.015). Table 2 shows the various co-morbidities in Group A and Group B. No case of osteoporosis was observed among patients without periodontitis, whereas 5.8% among those with periodontitis. The difference was statistically significant (p = 0.0001). In cases of diabetes mellitus, cardiovascular disorders (CVD), musculoskeletal disorders, respiratory disorders, hypertension, gastric disorders, psychiatric disorders, hypothyroidism, headache, rheumatoid arthritis, and COVID-19 infection, the prevalence was higher in the periodontitis group. However, the difference was not statistically significant. The prevalence of PCOS and ENT was higher in the non-periodontitis group. However, the difference was not statistically significant. The prevalence of allergies, liver disease, inflammatory bowel disease, kidney, and depression did not differ significantly between the two groups.

In Group B (periodontitis), 17.5% of patients aged 20-30, 22.5% among 31-40, and 27.5% in 41-55 years had comorbidities, as shown in Table 3. There was no statistically significant difference in the number of comorbidities with different age groups in periodontitis patients (p=0.051). In Group B (periodontitis), among males, 21.0%, and in females 40.2% cases had comorbidities. There was a statistically significant difference in the number of comorbidities (p=0.001). Among patients classified as underweight based on BMI, 0% had comorbidities. In the healthy category, 20.3% had comorbidities. For the overweight category, 40.8% had comorbidities. For the obese category, 100.0% had comorbidities. There was a statistically significant difference in the number of comorbidities according to BMI in periodontitis patients (p<0.0001). There was no statistically significant difference in the number of comorbidities according to the occupation (p=0.167) and various habits (p=0.508) in either group.

Table 4 shows that among periodontitis patients with excellent plaque levels, none had comorbidities. In the good plaque levels, 18.4% cases in fair plaque levels, 35% in poor plaque levels and 52.9% cases had comorbidities. There was a statistically significant difference in the number of comorbidities according to plaque level in periodontitis patients (p=0.002). Among patients with no periodontal disease or mild clinical attachment loss (CAL), 0.0%; in the moderate, 18.2%, and for severe CAL, 34.4% cases had comorbidities. There was a statistically significant difference in the number of comorbidities according to CAL in periodontitis patients (p=0.010). Regarding PBI in periodontitis patients, in the very good category, 23.7%; in the good, 50.0%, and in the poor PBI category, 50.0% of cases had comorbidities. There was a statistically significant difference in PBI between with and without comorbidities in periodontitis patients, (p=0.001). Group B (periodontitis), among patients with excellent plaque levels category, 0.0%, in good plaque levels; 18.4%, in fair plaque levels, 35.0%, and with poor plaque levels, 52.9% cases had comorbidities. There was a statistically significant difference in the number of comorbidities according to plaque level (p=0.002)

## Discussion:

In this study, each patient filled out medical health questionnaires containing relevant questions about systemic diseases, verified by an interview with a dental professional to reduce the risk of bias. The validity of self-reported questionnaires has been investigated and has shown a good degree of validity. [11][12] In this study, Periodontitis patients with comorbidities were found to be more than the non-periodontitis group, though it was not statistically significant (p=0.09). However, the number of comorbidities in periodontitis patients was found to be statistically significantly more than comorbidities in non-periodontitis patients. The difference in plaque indices and papillary bleeding index in the periodontitis group with co-morbidities was statistically significantly higher than in patients without co-morbidities. Thus, Poor oral hygiene increases the risk of the onset of systemic diseases. [13] The mean clinical attachment loss in the periodontitis group with co-morbidities was higher than in periodontitis patients without co-morbidities, and the result was statistically significant (p= 0.010). This revealed a linear pattern between the severity of periodontitis and the presence of one or more comorbidities. Periodontopathic bacteria trigger a local inflammatory response causing systemic effects that negatively affect the inflammatory disorders. [14] Results showed that there was a higher prevalence of osteoporosis among patients with periodontitis. Locally increased production of cytokines such as kappa β-ligand (RANKL), tumor necrosis factor (TNF-α), IL-6, and IL-1β by immune cells and osteoclasts in periodontitis can accelerate systemic bone resorption. [15] Many researchers have proposed menopause as a risk factor for osteoporosis which is related to reduced estrogen, as more comorbidities seen in this study were found in female periodontitis patients. [16]

The prevalence of diabetes mellitus, CVD, and hypertension was found to be higher in the periodontitis group than in the non-periodontitis group, though the difference was non-significant. Similarly, a study in the Swedish population reported 44.3% of patients with CVD and 21.2% of diabetes mellitus in periodontitis patients which was significantly higher than in non-periodontitis patients. [17] Their sample size was 325 periodontitis patients, comparable to this study. But the median age taken was higher i.e., 62 years, as compared to the median age of 37 years in this study; indicating that increased age could be the reason for finding a higher prevalence of CVD and diabetes in their study. Also, they had included hypertension under CVD, whereas it was taken separately in this study. There was no statistically significant difference in COVID-19 infection between the two groups, (p=0.300). No case of severe COVID complications was observed. The results were in contrast to a study, in which periodontitis was strongly associated with COVID-19 infection and three times more COVID-19 complications. [18] Considering the BMI of patients with periodontitis, results suggested a significant association between obesity and comorbidities in periodontitis patients (p<0.0001). Tumor necrosis factor-α (TNF-α), one of many pro-inflammatory cytokines produced in periodontitis could be a crucial inflammatory cytokine encouraging obesity.[19] Therefore, dentists should be aware that overweight and obese periodontal patients should maintain good oral hygiene practices, as periodontitis can be considered as one of the causal factors of obesity. The outcome of this research disclosed that periodontitis increased the risk for various systemic diseases.

## Conclusion:

Co-morbidity was prevalent in the periodontitis group (Group B). Poor oral hygiene with a higher plaque index, higher papillary bleeding index, increased clinical attachment loss and obesity showed a positive correlation with the increased number of comorbidities among periodontitis patients.

## Figures and Tables

**Table 1:** Comparison of Co-morbidities and Number of Co-morbidities between Group A and Group B; *p-value < 0.05

**Comorbidities**		**Group A**	**Group B**	**P value**
		Non-periodontitis	Periodontitis	
		Column N %	Column N %	
	No	77.50%	70.80%	0.095
	Yes	22.50%	29.20%	
**Number of Comorbidities**		**Group A**	**Group B**	
		Non-periodontitis	Periodontitis	0.015*
		Column N %	Column N %	
	No	67.90%	58.00%	
	Yes	32.10%	42.00%	

**Table 2:** Cross-tabulation of Co-morbidities between Group A and Group B; *p-value < 0.05

**Comorbidities**	**Group A**	**Group B**	**P value**
	**(Non-periodontitis)**	**(Periodontitis)**	
	**Percentage**	**Percentage**	
Diabetes Mellitus	1.70%	4.60%	0.07
Cardiovascular disorders	0.40%	0.80%	0.56
Muscular skeletal disorders	2.50%	3.80%	0.43
Respiratory disorders	0.40%	0.80%	0.56
Hypertension	2.90%	3.30%	0.79
Osteoporosis	0.00%	5.80%	0.0001*
Cancer/Oncology	0.00%	0.00%	n/a
Allergies	1.30%	1.30%	1
Gastric disorders	4.20%	5.40%	0.52
Psychiatric disorders (excluding depression)	0.00%	0.40%	0.32
Liver diseases	0.40%	0.40%	1
Polycystic ovarian syndrome (PCOS)	1.70%	0.80%	0.41
Neurological disorders	0.00%	0.00%	n/a
Inflammatory bowel disease	0.80%	0.80%	1
Hypothyroidism	0.80%	2.10%	0.25
Headache	1.30%	2.10%	0.48
Eye disease	0.40%	0.40%	1
ENT disease	0.80%	0.00%	0.16
Kidney diseases	0.40%	0.40%	1
Rheumatoid arthritis	0.00%	0.80%	0.16
Depression	0.40%	0.40%	1
COVID-19 complications	9.20%	12.10%	0.3

**Table 3:** Comparison of Age, Gender, Body mass index, Occupation, and Habits (Alcohol consumption and smoking) in Group B with & without comorbidities *p-value < 0.05

		**Comorbidities**		
		**No**	**yes**	**P value**
		**Row N %**	**Row N %**	
Age	20-30	82.50%	17.50%	0.318
	31-40	77.50%	22.50%	
	41-55	72.50%	27.50%	
		Comorbidities		
		No	Yes	
		Row N %	Row N %	0.001*
Sex	Male	79.00%	21.00%	
	Female	59.80%	40.20%	
		Comorbidities		0.0001*
		No	Yes	
		Row N %	Row N %	
BMI	Under weight	100.00%	0.00%	
	Healthy	79.70%	20.30%	
	Overweight	59.20%	40.80%	
	Obese	0.00%	100.00%	
		Comorbidities		0.167
		No	Yes	
		Row N %	Row N %	
Occupation	Unemployed	60.50%	39.50%	
	Unskilled worker	80.00%	20.00%	
	Semi-skilled worker	100.00%	0.00%	
	Skilled worker	73.10%	26.90%	
	Clerical, shop-owner/farm	69.80%	30.20%	
	Semi-professional	82.40%	17.60%	
	Professional	78.60%	21.40%	
		Comorbidities		
		No	Yes	
		Row N %	Row N %	
				0.508
	No	71.90%	28.10%	
Habit				
	Yes	67.30%	32.70%	

**Table 4:** Comparison of Plaque index, Clinical attachment loss (CAL) and Papillary bleeding index (PBI) in Group B with & without comorbidities; *p- value < 0.05

		**Comorbidities**		**P value**
		**No**	**Yes**	
		**Row N %**	**Row N %**	
Plaque	Excellent	0.00%	0.00%	0.002*
	Good	81.60%	18.40%	
	Fair	65.00%	35.00%	
	Poor	47.10%	52.90%	
		Comorbidities		0.010*
		No	Yes	
		Row N %	Row N %	
CAL	No PD/ Mild	0.00%	0.00%	
	Moderate	81.80%	18.20%	
	Severe	65.60%	34.40%	
		Comorbidities		0.001*
		No	Yes	
		Row N %	Row N %	
PBI	Very good	76.30%	23.70%	
	Good	50.00%	50.00%	
	Poor	50.00%	50.00%	
